# Isorhynchophylline Protects PC12 Cells Against Beta-Amyloid-Induced Apoptosis via PI3K/Akt Signaling Pathway

**DOI:** 10.1155/2013/163057

**Published:** 2013-11-06

**Authors:** Yan-Fang Xian, Zhi-Xiu Lin, Qing-Qiu Mao, Jian-Nan Chen, Zi-Ren Su, Xiao-Ping Lai, Paul Siu-Po Ip

**Affiliations:** ^1^School of Chinese Medicine, Faculty of Medicine, The Chinese University of Hong Kong, Shatin, Hong Kong; ^2^College of Chinese Medicines, Guangzhou University of Chinese Medicine, 510006 Guangzhou, China

## Abstract

The neurotoxicity of amyloid-**β** (A**β**) has been implicated as a critical cause of Alzheimer's disease. Isorhynchophylline (IRN), an oxindole alkaloid isolated from *Uncaria rhynchophylla, *exerts neuroprotective effect against A*β*_25–35_-induced neurotoxicity *in vitro*. However, the exact mechanism for its neuroprotective effect is not well understood. The present study aimed to investigate the molecular mechanisms underlying the protective action of IRN against A*β*_25–35_-induced neurotoxicity in cultured rat pheochromocytoma (PC12) cells. Pretreatment with IRN significantly increased the cell viability, inhibited the release of lactate dehydrogenase and the extent of DNA fragmentation in A*β*_25–35_-treated cells. IRN treatment was able to enhance the protein levels of phosphorylated Akt (p-Akt) and glycogen synthase kinase-3**β** (p-GSK-3**β**). Lithium chloride blocked A*β*_25–35_-induced cellular apoptosis in a similar manner as IRN, suggesting that GSK-3**β** inhibition was involved in neuroprotective action of IRN. Pretreatment with LY294002 completely abolished the protective effects of IRN. Furthermore, IRN reversed A*β*_25–35_-induced attenuation in the level of phosphorylated cyclic AMP response element binding protein (p-CREB) and the effect of IRN could be blocked by the PI3K inhibitor. These experimental findings unambiguously suggested that the protective effect of IRN against A*β*_25–35_-induced apoptosis in PC12 cells was associated with the enhancement of p-CREB expression via PI3K/Akt/GSK-3**β** signaling pathway.

## 1. Introduction

Alzheimer's disease (AD) is the most common form of neurodegenerative disorders of the brain and affects an estimated 26.6 million people across the globe in 2006 [1]. The neuropathological hallmarks of AD include massive accumulation of beta-amyloid (A*β*) in senile plaques, abnormal tau filaments in neurofibrillary tangles, and extensive neuronal loss [2, 3]. A*β* is a 39- to 43-amino acid peptide fragment derived from sequential proteolysis of amyloid precursor protein (APP) through cleavage by *β*-secretase and *γ*-secretase [4]. Recent studies have suggested that A*β* plays an important role in the pathogenesis of AD [5]. A*β* accumulation has been causatively implicated in the neuronal dysfunction and neuronal loss that underlie the clinical manifestations of AD [6]. A correlation among memory deficits, A*β* elevation, and amyloid plaques on transgenic has been reported in previous studies [7, 8]. Therefore, inhibition of A*β*-induced neuronal degeneration may provide clinical benefits to AD patients.

**Figure 1:**
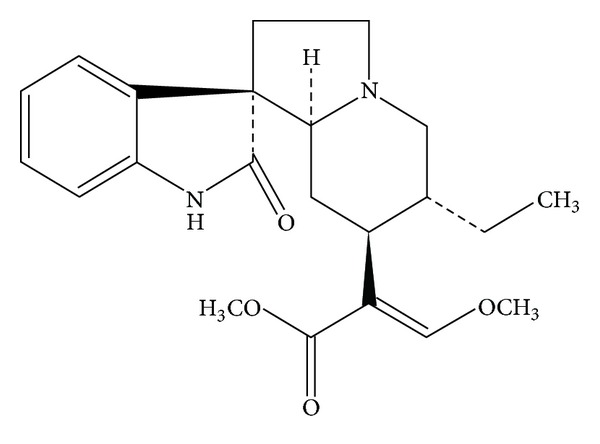
Chemical structure of isorhynchophylline (IRN).

Isorhynchophylline (IRN, Figure 1), an oxindole alkaloid, has been identified as the main active ingredient responsible for the biological activities of *Uncaria rhynchophylla *[9, 10]. IRN has also been reported to protect against the ischemia- and glutamate-induced neuronal damage or death [9, 11], and inhibition of 5-HT receptor [12, 13]. Previous studies in our laboratory has demonstrated that IRN protected rat pheochromocytoma (PC12) cells against the A*β*_25–35_-induced oxidative stress, mitochondrial dysfunction, apoptosis, calcium influx, and tau protein hyperphosphorylation [14, 15]. However, the molecular mechanisms underlying the protective effect of IRN against the neurotoxicity induced by A*β*_25–35_ have not been fully understood. In this study, we aimed to elucidate the molecular signaling pathway involved in the neuroprotective effect of IRN.

## 2. Materials and Methods

### 2.1. Chemicals and Reagents

Isorhynchophylline (IRN, purity ⩾ 98%) was purchased from Chengdu Mansite Pharmaceutical Co. Ltd. (Chengdu, Sichuan, China). Its identity was confirmed by comparing its H^1^ NMR spectra with the published data [16]. Nerve growth factor (NGF), LY294002 (LY), lithium chloride (LiCl), and *β*-amyloid peptide (A*β*_25–35_) were purchased from Sigma-Aldrich (St. Louis, MO, USA). Dulbecco's modified Eagle medium (DMEM), fetal bovine serum (FBS), penicillin, and streptomycin were obtained from Gibco (Grand Island, NY, USA). Unless otherwise indicated, all other reagents were of analytical grade and were obtained from Sigma-Aldrich.

### 2.2. Peptide Preparation

A*β*_25–35_, which is the most toxic peptide fragment derived from amyloid precursor protein, was dissolved in deionized distilled water at the concentration of 1 mM. The stock solution was diluted to desired concentrations immediately before use and added to cell culture medium.

### 2.3. Cell Culture and Drug Treatment

The PC12 cells were obtained from the American Type Culture Collection (Rockville, MD, USA). They were maintained in DMEM medium supplemented with penicillin (100 U/mL), streptomycin (100 *μ*g/mL), 6% FBS, and 6% horse serum at 37°C in a humidified atmosphere of 95% air and 5% CO_2_. Unless otherwise specified, the cells were seeded onto 24-well culture plate at a density of 8 × 10^4^ cells/well. PC12 cells were differentiated with 50 ng/mL NGF in serum-free DMEM for 3 days [15]. IRN and all inhibitors were dissolved in DMSO and diluted with culture medium. The final concentration of DMSO in the test solutions was less than 0.1%. The cells were incubated with different concentrations of IRN (final concentrations: 1, 10, and 50 *μ*M) for 2 h. A*β*_25–35_ at a final concentration of 20 *μ*M was then added to the culture for an additional 24 h. In experiments involving kinase inhibitors, the inhibitors LY294002 (50 *μ*M) or LiCl (10 mM) were added 1 h prior to IRN (50 *μ*M) and/or A*β*_25–35_ (20 *μ*M) treatment.

### 2.4. Cell Viability Assay

Cell viability was measured using a CellTiter 96 AQ_ueous_ One Solution Cell Proliferation Assay (Promega, Madison, WI, USA). In brief, PC12 cells were seeded onto a 96-well culture plate at a density of 2 × 10^4^ cells/well. Cells were washed with D-Hanks solution after drug treatment. Then, 100 *μ*L of serum-free medium and 20 *μ*L of CellTiter 96 AQ_ueous_ One Solution were added into each well. The cells were incubated at 37°C for 2 h. The quantity of formazan product, which is directly proportional to the number of living cells, was measured using a FLUOstar OPTIMA microplate reader (BMG Labtech, Offenbury, Germany) at 490 nm. Cell viability was expressed as percentage of nontreated control.

### 2.5. Lactate Dehydrogenase (LDH) Activity Assay

LDH activity was measured using a LDH diagnostic kit (STANBIO Laboratory, Boerne, TX, USA) according to the manufacturer's protocol. Briefly, PC12 cells were seeded onto 24-well culture plates at a density of 1 × 10^5^ cells/well. At the end of the drug treatment, the medium was collected. Subsequently, 100 *μ*L of the medium was added to a polystyrene cuvette containing 1 mL of LDH reagent. The cuvette was placed immediately into a spectrophotometer and maintained at 30°C. After stabilization for 1 min, the absorbance at 340 nm was recorded at 1 min intervals for 3 min. The enzyme activity was expressed in unit per liter. To determine intracellular LDH activity, the cells were washed with D-Hanks solution and then scraped from the plates into 500 *μ*L of ice-cold PBS (0.1 M, containing 0.05 mM of EDTA) and homogenized. The homogenate was centrifuged (4000 ×g) at 4°C for 30 min. The resulting supernatant was collected for the LDH assay. The total LDH activity was computed by summing the activities in the cell lysate and medium. Cellular toxicity was indicted by the percentage of LDH released from the cell.

### 2.6. Quantification of DNA Fragmentation

Quantification of DNA fragmentation was determined by Cell Death Detection ELISA^Plus^ kit (Roche Applied Sciences, Basel, Switzerland) according to the manufacturer's protocol. In brief, the cells were washed with HBSS after drug treatment. Then, the cells were incubated with 200 *μ*L of lysis buffer for 30 min at room temperature. The plate was centrifuged at 200 ×g for 10 min at 4°C. An aliquot (20 *μ*L) of the supernatant from each well was transferred into a streptavidin-coated microplate and incubated with a mixture of anti-histone biotin and anti-DNA peroxidase. The apoptotic nucleosomes were captured via their histone component by the anti-histone-biotin antibody which was bound to the streptavidin-coated microplate. Simultaneously, anti-DNA peroxidase was bound to the DNA part of the nucleosomes. After removing the unbound antibodies, the amount of peroxidase retained in the immunocomplex was quantified by adding 2,2′-azinobis (3-ethylbenzthiazoline-6-sulphonic acid) (ABTS) as the substrate, and the absorbance of the reaction mixture was measured at 405 nm using a microplate reader. The absorbance is directly proportional to the number of apoptotic nucleosomes. The extent of DNA fragmentation was expressed as percentage of the control.

### 2.7. Western Blot Analysis

PC12 cells were seeded onto 100 mm^2^ dish at 5 × 10^6^ cells/dish. The cells were washed twice with D-Hanks solution after drug treatment. The cells were harvested and lysed with protein lysis buffer (50 mM Tris-HCl, pH 7.5, 100 mM NaCl, 1% NP-40, 0.5% sodium deoxycholate, 0.1% SDS, 1 mM EDTA, 1 mM sodium orthovanadate, 10 mM sodium fluoride, and 100 mg/mL PMSF). Protein concentration in the supernatants was determined with the BCA protein assay. Protein samples were electrophoresed by SDS-PAGE for 2 h at 80 V. The separated proteins were transferred to polyvinylidene fluoride (PVDF) membranes using a transblotting apparatus (Bio-Rad Laboratories, Hercules, CA, USA) for 30 min at 15 V. The membranes were blocked with 5% (w/v) nonfat milk in TBS-T (Tris-buffer saline containing 0.1% Tween-20) at room temperature for 2 h and subsequently incubated at 4°C overnight with appropriate amount of primary antibodies against p-Akt (Ser 473), Akt, phosphorylation of glycogen synthase kinase-3*β* (p-GSK-3*β*, Ser9), GSK-3*β*, phosphorylation cyclic AMP response element binding protein (p-CREB, Ser133), CREB (Cell Signaling Technology, Beverly, MA), and *β*-actin (Santa Cruz Biotechnology Inc., USA) at 4°C overnight. Next, the membrane was washed with TBS-T three times and probed with horseradish peroxidase conjugated secondary antibody at room temperature for 1 h. To verify equal loading of samples, the membranes were incubated with monoclonal antibody *β*-actin, followed by a horseradish peroxidase conjugated goat anti-mouse IgG. The membrane again was washed with TBS-T for three times, and finally the protein bands were visualized by the ECL western blotting detection reagents (Amersham Biosciences, Buckinghamshire, UK). The intensity of each band was analyzed using Image J software (NIH Image, Bethesda, MD, USA).

### 2.8. Statistical Analysis

Data were expressed as mean ± SEM. Multiple group comparisons were performed using one-way analysis of variance (ANOVA) followed by Tukey's test in order to detect intergroup differences. GraphPad Prism software (Version 4.0; GraphPad Software, Inc., San Diego, CA) was used to perform the statistical analysis. A difference was considered statistically significant if the *P* value was less than 0.05.

## 3. Results

### 3.1. Effects of IRN on A*β*_25–35_-Induced Cytotoxicity in PC12 Cells

**Figure 2:**
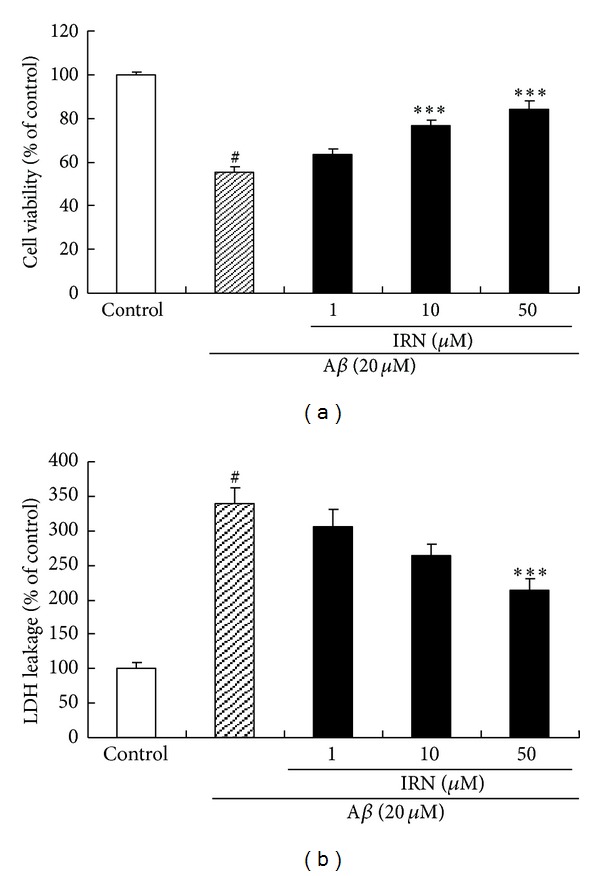
Effects of IRN on the A*β*_25–35_-induced neurotoxicity in PC12 cells. Cell viability was measured by MTS assay (a) and LDH assay (b). Values given are the mean ± SEM (*n* = 6). ^#^ *P* < 0.001 compared with the control group; ***P < 0.001 compared with the A*β*_25–35_-treated control.

The effect of IRN on cell viability of A*β*_25–35_-treated PC12 cells was shown in Figure 2(a). Treating the cells with A*β*_25–35_ at 20 *μ*M for 24 h could significantly decrease cell viability, as compared to the control group (*P* < 0.001). Pretreatment with IRN (10 and 50 *μ*M) in the presence of 20 *μ*M A*β*_25–35_ for 24 h was able to significantly increase the cell viability as compared with the A*β*_25–35_-treated control (*P* < 0.001 for both concentrations).

To investigate the protective effect of IRN, a LDH assay was performed. As shown in Figure 2(b), when PC12 cells were incubated with 20 *μ*M of A*β*_25–35_ for 24 h, the percentage of LDH leakage was conspicuously increased (*P* < 0.001). When the cells were pretreated with IRN (50 *μ*M) in the presence of 20 *μ*M of A*β*_25–35_ for 24 h, the percentage of LDH leakage was significantly reduced as compared with the A*β*_25–35_-treated control (*P* < 0.001).

### 3.2. Effect of IRN on A*β*_25–35_-Induced Activation of GSK-3*β* in PC12 Cells

**Figure 3:**
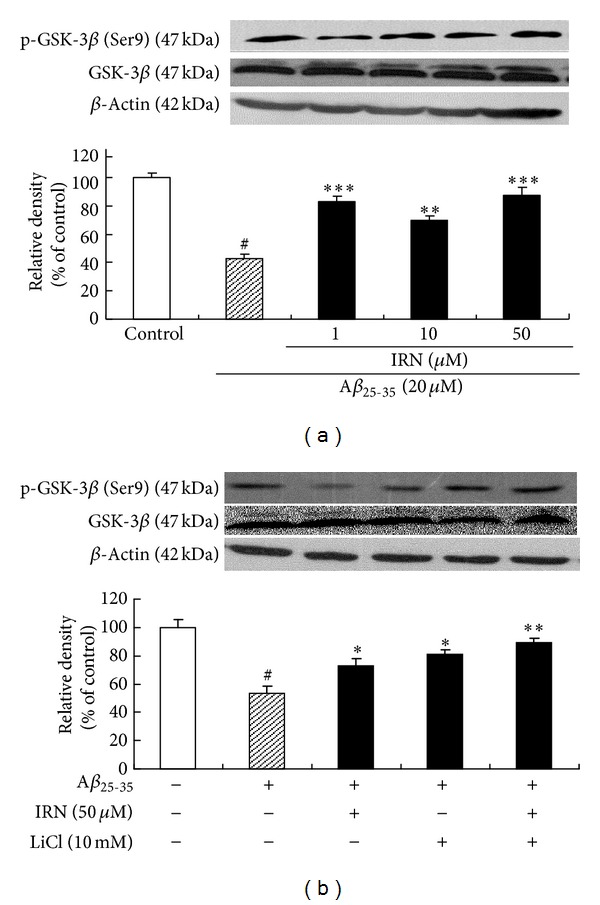
Effects of IRN on the A*β*_25–35_-induced activation of GSK-3*β* in PC12 cells. Values given are the mean ± SEM (*n* = 3). ^#^ *P* < 0.001 compared with the control group; *P < 0.05, **P < 0.01, and ***P < 0.001 compared with the A*β*_25–35_-treated control.

To investigate the effect of IRN on the activation of GSK-3*β* in A*β*_25–35_-treated PC12 cells, the protein levels of GSK-3*β* and p-GSK-3*β* (Ser9) were determined. As shown in Figure 3(a), the level of p-GSK-3*β* was significantly decreased (*P* < 0.001) after treatment with 20 *μ*M of A*β*_25–35_. Interestingly, pretreatment with IRN (1, 10 and 50 *μ*M) markedly elevated the level of p-GSK-3*β* (*P* < 0.00, *P* < 0.01 and *P* < 0.001, resp.) when compared to the A*β*_25–35_-treated control, indicating that IRN suppressed the activation of GSK-3*β* induced by A*β*_25–35_. To show the correlation between p-GSK-3*β* and cell viability, LiCl, a potent GSK-3*β* inhibitor, was used. Results showed that pretreatment with LiCl (10 mM) could significantly accentuate cell viability (*P* < 0.01, Figure 6(a)) and the protein level of p-GSK-3*β* (*P* < 0.05, Figure 3(b)). The treatment also attenuated LDH leakage (*P* < 0.001, Figure 6(b)) and DNA fragmentation (*P* < 0.001, Figure 6(c)) in A*β*_25–35_-treated cells.

### 3.3. Effect of IRN on A*β*_25–35_-Induced Inactivation of PI3K/Akt Pathway

**Figure 4:**
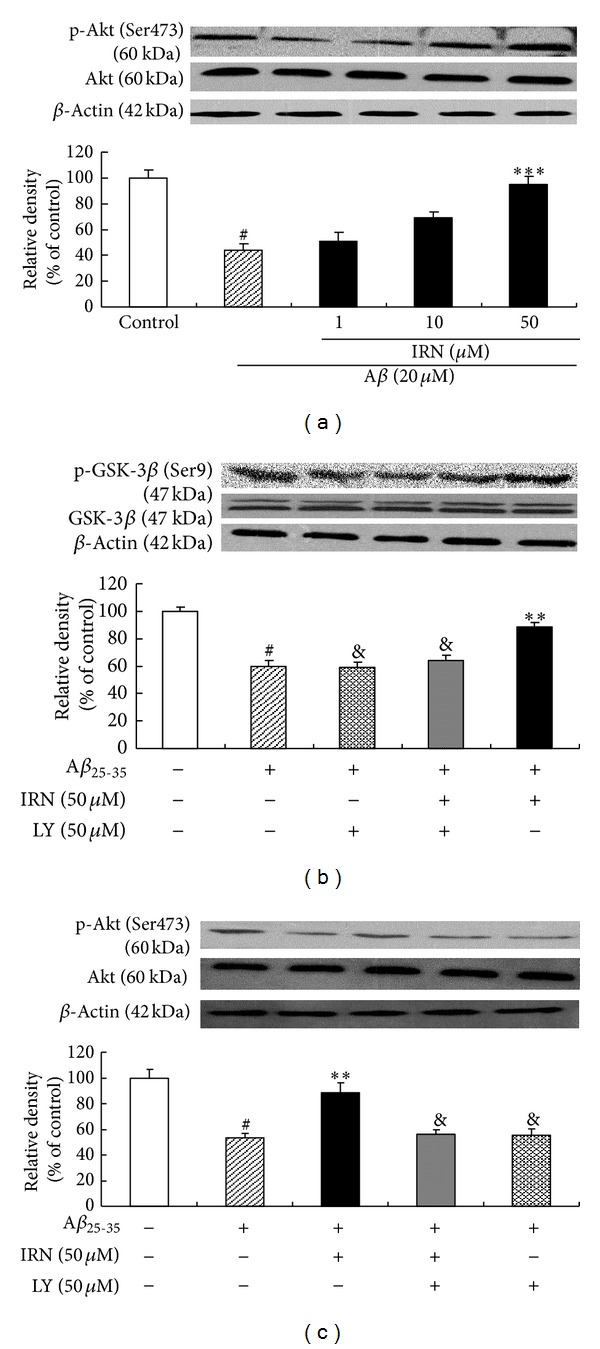
Effect of IRN on A*β*_25–35_-induced inactivation of PI3K/Akt pathway. Values given are the mean ± SEM (*n* = 3). ^#^ *P* < 0.001 compared with the control group; **P < 0.01 and ***P < 0.001 compared with the A*β*_25–35_-treated control; ^&^ *P* < 0.05 compared with the group treated with A*β*_25–35_ and IRN (50 *μ*M).

As shown in Figure 4(a), treatment with 20 *μ*M of A*β*_25–35_ for 24 h significantly decreased the protein level of p-Akt (Ser473). However, pretreatment with IRN (50 *μ*M) markedly increased the protein level of p-Akt (*P* < 0.001), indicating that IRN was able to activate PI3K/Akt signaling pathway in the A*β*_25–35_-treated cells. LY294002, a potent PI3K/Akt signaling pathway inhibitor [17, 18], thoroughly abolished the effects of IRN on p-Akt and p-GSK-3*β* (Figures 4(b) and 4(c)). In these experiments, total protein levels of Akt and GSK-3*β* did not change in all groups.

### 3.4. Effect of IRN on the Phosphor-CREB through PI3K Activation

**Figure 5:**
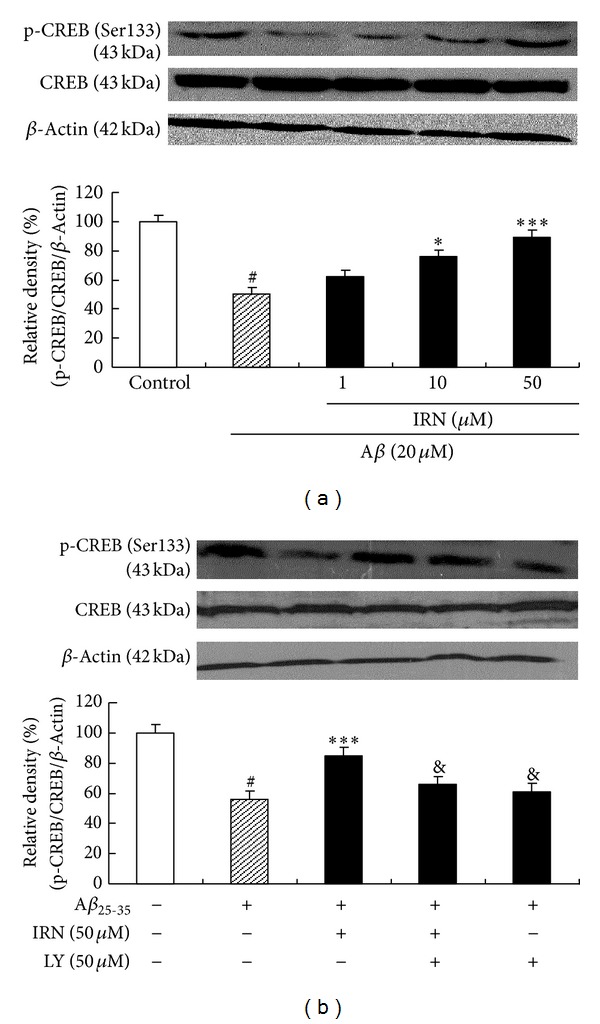
Effect of IRN on the p-CREB through PI3K activation. Values given are the mean ± SEM (*n* = 3). ^#^ *P* < 0.001 compared with the control group; *P < 0.05 and ***P < 0.001 compared with the A*β*_25–35_-treated control; ^&^ *P* < 0.05 compared with the group treated with A*β*_25–35_ and IRN (50 *μ*M).

As shown in Figure 5, treating the cells with 20 *μ*M of A*β*_25–35_ for 24 h significantly decreased protein level of p-CREB (Ser133), while pretreatment with IRN (10 and 50 *μ*M) markedly elevated protein level of p-CREB (*P* < 0.05 and *P* < 0.001, resp.), as compared with the A*β*_25–35_-treated control. The effect of IRN was completely blocked by LY294002, indicating the involvement of PI3K signal transduction.

## 4. Discussion

Previous studies in our laboratory demonstrated that IRN could significantly reduce the neurotoxicity induced by A*β*_25–35_ via inhibiting oxidative stress, calcium influx, tau protein hyperphosphorylation, and suppressing cellular apoptosis in PC12 cells [14, 15]. The present study revealed that IRN could protect PC12 cells against the A*β*_25–35_-induced neurotoxicity via PI3K/Akt/GSK-3*β* signaling pathway.

**Figure 6:**
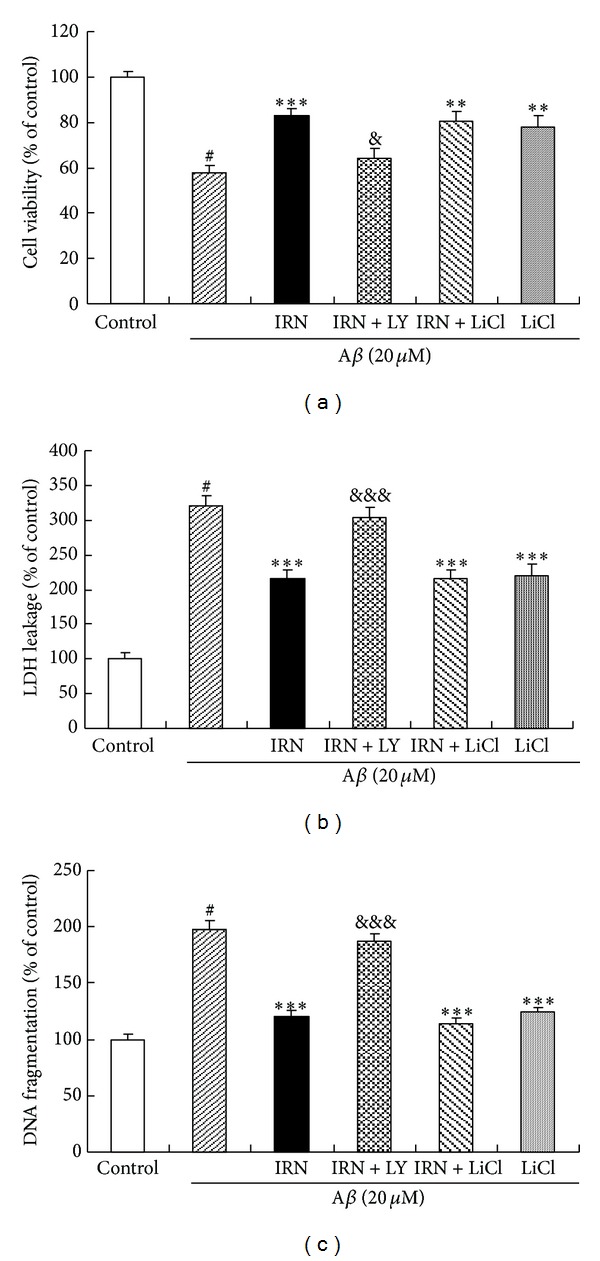
Effect of kinase inhibitors on neuroprotection of IRN against A*β*_25–35_-induced neurotoxicity. A*β*_25–35_-induced neurotoxicity was indicated by cell viability (a), LDH leakage (b), and the production of DNA fragmentation (c), respectively. Values given are the mean ± SEM (*n* = 6). ^#^ *P* < 0.001 compared with the control group; **P < 0.01 and ***P < 0.001 compared with the A*β*_25–35_-treated control; ^&^ *P* < 0.05 and ^&&&^ *P* < 0.001 compared with the group treated with A*β*_25–35_ and IRN (50 *μ*M).

Recent studies suggest that GSK-3*β* plays an important role in AD neuropathology [19] and its activity accounts for many pathological hallmarks of the disease in both sporadic and familial AD cases. Hyperactivation of GSK-3*β* has been reported to induce neuronal cell death [20] and abnormal tau protein hyperphosphorylation [21, 22], both of which are the cardinal pathogenesis of AD. GSK-3*β* genes have been identified as potential candidate susceptibility genes for dementia [23]. In addition, GSK-3*β* expression is elevated in APP transgenic cultures which is coincided with the development of neuronal injury in brains of AD patients [24, 25]. Unlike most protein kinases, phosphorylation of GSK-3*β* at Ser9 leads to the inactivation of the enzyme [26]. Therefore, upregulation of p-GSK-3*β* (Ser9) may confer a protective effect. Our findings showed that A*β* significantly reduced the protein level of p-GSK-3*β* (Ser9), while pretreatment with IRN variably elevated the protein level of p-GSK-3*β* (Ser9). Treating the cells with LiCl, an inhibitor of GSK-3*β*, produces similar effects as IRN on A*β*_25–35_-induced cytotoxicity (Figure 6). However, synergistic effect was not observed between IRN and LiCl. Our previous studies demonstrated that IRN was able to reverse cellular apoptosis and tau protein hyperphosphorylation in A*β*-treated PC12 cells [14, 15]. These results suggest that the protective effect of IRN against A*β*_25–35_-induced apoptosis and tau protein hyperphosphorylation may be mediated by the suppression of GSK-3*β* activation.

Akt is a well-known prosurvival kinase and is activated by the phosphorylation at the Ser473 via PI3K pathway [27, 28]. PI3K/Akt signaling pathway has been suggested to play a pivotal role in GSK-3*β*-mediated tau protein hyperphosphorylation and neuronal survival. Inhibition of PI3K/Akt signaling pathway increases GSK-3*β* activity, resulting in tau protein hyperphosphorylation [29]. PI3K enhances neuroprotection through regulating phosphorylation level and activation of the Akt. Akt activity can be modulated by phosphorylation either on the residue Thr308 or Ser473 [30, 31]. The activation of Akt can lead to the suppression of GSK-3*β* activity [32]. Our results showed that A*β* treatment significantly decreased the protein level of p-Akt (Ser473) in PC12 cells. Pretreatment with IRN could significantly reverse the effect of A*β* on p-Akt which accounted for the protective mechanism of IRN against A*β*-induced neurotoxicity. Cotreatment with LY294002, a specific inhibitor of PI3K, completely abolished the effect of IRN on p-Akt and p-GSK-3*β* (Figure 4). Consistent results were obtained for the interaction of LY294002 and IRN on A*β*-induced cytotoxicity and DNA fragmentation (Figure 6). These findings strongly suggested that the protective effect of IRN against the A*β*_25–35_-induced neurotoxicity in PC12 cells was medicated via PI3K/Akt signaling pathway.

In addition to robustly phosphorylated tau protein, GSK-3*β* also acts as a key regulator of a broad array of transcriptional factors, that is, *β*-catenin, activator protein-1, nuclear factor kappa B (NF*κ*B), p53, CREB, heat shock factor (HSF-1), and CCAAT/enhancer binding protein [33]. Among these factors, CREB is the most important element in regulating cell survival and death. P-CREB (Ser133) is a downstream protein of the PI3K/Akt pathway [34] and acts as a substrate for GSK-3*β* [35]. It participates in many vital processes, including cell survival [36]. Our data showed that treatment with A*β*_25–35_ markedly inhibited the content of p-CREB, while pretreatment with IRN significantly increased the content of p-CREB via PI3K activation. Recent studies revealed that Akt and CREB could promote cell survival by upregulating the expression of antiapoptotic proteins such as Bcl-2 [37, 38]. Interestingly, our previous study indicated that pretreatment with IRN could significantly enhance the expression of Bcl-2 [14]. Furthermore, our results showed that PI3K inhibitor, LY294002, could abolish the accentuating effect of IRN on the protein expression of p-CREB, suggesting that p-CREB was involved in the neuroprotective mechanism of IRN.

In summary, our results demonstrated that IRN could protect against the A*β*_25–35_-induced apoptosis in PC12 cells. The protective effect of IRN was associated with the enhancement of p-CREB expression via PI3K/Akt/GSK-3*β* signaling pathway. The results from the present study advance our knowledge regarding the neuroprotective mechanism of IRN. More importantly, this study has laid a foundation for future clinical studies to evaluate the potential benefits of IRN on AD patients.
